# Aggregate-driven reconfigurations of carbon nanotubes in thin networks under strain: *in-situ* characterization

**DOI:** 10.1038/s41598-019-41989-2

**Published:** 2019-04-02

**Authors:** Laurence Bodelot, Luka Pavić, Simon Hallais, Jérôme Charliac, Bérengère Lebental

**Affiliations:** 1 Ecole Polytechnique, Laboratoire de Mécanique des Solides (LMS), 91128 Palaiseau, France; 2 Ecole Polytechnique, Laboratoire de Physique des Interfaces et Couches Minces (LPICM), 91128 Palaiseau, France; 3 Université Paris-Est, IFSTTAR, COSYS, Marne-La-Vallée, 77447 France; 4 Present Address: Division of Materials Chemistry, Ruđer Bošković Institute, Bijenička cesta 54, 10000 Zagreb, Croatia

## Abstract

This work focuses on the *in-situ* characterization of multi-walled carbon nanotube (CNT) motions in thin random networks under strain. Many fine-grain models have been devised to account for CNT motions in carbon nanotube networks (CNN). However, the validation of these models relies on mesoscopic or macroscopic data with very little experimental validation of the physical mechanisms actually arising at the CNT scale. In the present paper, we use *in-situ* scanning electron microscopy imaging and high-resolution digital image correlation to uncover prominent mechanisms of CNT motions in CNNs under strain. Results show that thin and sparse CNNs feature stronger strain heterogeneities than thicker and denser ones. It is attributed to the complex motions of individual CNTs connected to aggregates within thin and sparse CNNs. While the aggregates exhibit a collective homogeneous deformation, individual CNTs connecting them are observed to fold, unwind or buckle, seemingly to accommodate the motion of these aggregates. In addition, looser aggregates feature internal reconfigurations via cell closing, similar to foam materials. Overall, this suggests that models describing thin and sparse CNN deformation should integrate multiphase behaviour (with various densities of aggregates in addition to individual CNTs), heterogeneity across surface, as well as imperfect substrate adhesion.

## Introduction

Due to their large specific surface area^1^, carbon nanotubes (CNTs) have been of utmost interest for sensing applications since the early days of CNT research^2,3^. Electronic devices using CNTs have demonstrated exceptional sensitivity to their environment and have resulted in analytical^4^ (humidity, pH, gas, chemical or biological species), mechanical^5^ (strain, pressure) or radiation^6^ (thermal or infrared, UV) sensors. Among these examples, flexible strain sensors occupy a prominent place^7,8^ since they have shown promise for demanding applications such as human welfare^9^ or infrastructure^10,11^ monitoring. The mechanical robustness of CNTs (high Young’s modulus, low bending rigidity, low buckling properties, high tensile strength^2,12^) indeed provides enhanced device reliability. Although piezoresistive strain sensors with very high gauge factors (269 in Chang *et al*.^13^ and 600 to 1000 in Cao *et al*.^14^) may be achieved by exploiting the piezoresistivity of single-CNT devices, the most popular approach to CNT-based strain sensors relies on using carbon nanotube networks (CNNs)^15^. Three main categories of sensors are reported^7^: 3D bulk architectures (the so-called self-sensing materials)^16,17^, self-standing thin films (the so-called buckypapers)^18^ and substrate-supported thin films^9,19^. They are made either of pure CNTs (single-walled or multi-walled)^19^ or of CNT composites (namely, CNTs mixed with a matrix material such as a polymer^20^ or cement^16^).

To understand the mechanisms of piezoresistivity in CNNs, experimental resistance-strain curves are often fitted with detailed numerical models of the CNNs under strain (as in Feng and Jiang^21^, for instance). Usually, a microscopic scale Representative Volume Element (RVE) of a CNN is built numerically^22^ or reconstructed from processed SEM images of an actual CNN^23^. Hypotheses are first made regarding the electronic transport within the CNN (e.g., derivation of the tunnelling resistance between CNTs) as well as the behaviour of CNTs under strain (e.g., derivation of the strain dependence on the shortest path among CNTs)^21,24^. Then, the electrical and mechanical behaviours are calculated, for instance, by a Monte Carlo simulation^22^. These models match well with experimental results for low density CNNs (i.e., below or close to the electrical percolation threshold) in which the piezoresistivity is thought to be controlled by the change of the average inter-tube distance when the network is under strain^24^.

Above the percolation threshold, the piezoresistivity is usually attributed to a variation of the number of contacts when the network is under strain^25^. However, experimental results show that this proposed mechanism may not be fully accurate for denser CNNs: in Yin *et al*.^26^ for example, the authors suggest that the piezoresistivity of a CNN/polymer composite made out of long multi-walled carbon nanotubes (MWCNTs) may be mostly controlled by the presence of aggregates (areas with higher densities of strongly entangled CNTs). As a consequence, numerical models have now evolved to better account for CNT entanglements^27,28^ and surface-CNT interactions^29^ (not only for 3D and self-standing CNNs^27^, but also for supported CNNs^29^). They appear to predict well the global mechanical and electrical behaviour of the CNNs under study, including even complex phenomena such as buckling^27^ and piezoresistivity hysteresis^29^. The limitation of these approaches is that the relevance of the model is usually validated only by fitting global curves such as Young’s modulus or resistance as a function of strain. Until recently, there was very little proof that the microstructural features predicted by the models for CNNs under strain^27–29^—such as zipping, unzipping (separation of CNTs), reorientation, bundling or buckling—match well with the actual microstructural evolutions of the CNN.

In order to address this issue, *in-situ* scanning electron microscope (SEM) imaging of CNNs under strain has started to develop in recent years: in Hutchens *et al*.^30^ and Maschmann *et al*.^31^ very dense pillars of vertically-aligned CNTs show *in-situ* buckling under compressive strain; in Whitby *et al*.^32^ vortex-like motion of CNTs and pore diameter reduction is observed in a 3D dense CNN/polymer composite under compression; in Gui *et al*.^33^ the microstructural motion of a biphasic CNN is analysed under compression; in Abu Obaid *et al*.^34^ the evolution of the helicoidal organization of the fibres of CNT yarns is observed under tension; and in Stallard *et al*.^35^ straightening and buckling of fibres within direct-spun CNT mats submitted to very large tensile strains are reported. *In-situ* Raman and X-Ray scattering instead of SEM are used in Li *et al*.^36^ on a self-standing CNN and shows alignment of fibres according to the tensile strain axis. A prominent challenge of these methods is to provide a quantitative analysis of the images. Only Maschmann *et al*.^37^ have employed digital image correlation (DIC) to provide a mapping of compressive strains in the network—though at a resolution where CNTs are not resolved. So far, there is no *in-situ* SEM study on substrate-supported CNNs even though the microstructural understanding of the CNN-substrate interaction is critical to model these structures appropriately (as shown in Jin *et al*.^29^).

The present paper tackles this issue by studying, under *in-situ* SEM, the tensile response of inkjet-printed MWCNT films deposited on an ethylene tetrafluoroethylene (ETFE) substrate. Not only does the study address the impact of film thickness on the tensile response, but it also maps, for the first time, the strain fields at microscopic scale resolution using DIC. This mapping yields new insight into the motions of individual CNTs and CNT aggregates under strain. In particular, it is shown that strain heterogeneities in thin and sparse CNNs can be attributed to individual CNTs that fold, unwind or buckle to accommodate the motion of aggregates. Meanwhile, tight aggregates exhibit a collective homogenous deformation and looser aggregates feature internal reconfigurations via cell closing, similar to foam materials.

Note that due to electronic diffraction and charging effects, the fibre-like objects in SEM images of CNNs appear with overestimated diameters. In addition, small diameter CNTs packed within tight bundles usually cannot be resolved with SEM (a bundle is defined here, according to the literature^1^, as an ensemble of CNTs tightly packed along their length following a hexagonal lattice). As a consequence, the spatial resolution of our DIC-based methodology provides access to the motions of fibre-like objects in the SEM images. It does not, however, permit the observation of internal reorganization within these objects. Thus, it is not critical whether the fibres that are observed—referred to as “individual CNTs”—are actually single CNTs or tightly packed CNT bundles. The focus here lies on whether they behave mechanically as single elongated fibres that are well separated from their neighbours. By contrast, the internal reorganization of CNT aggregates (random assembly of CNT or CNT bundles) and CNT yarns^38^ (twisted ropes of CNT or CNT bundles) can be resolved by the proposed approach because individual fibres can be identified within their shape.

## Results and Discussion

**Figure 1:**
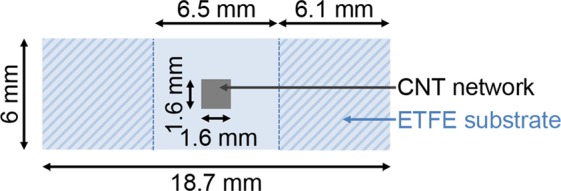
Top-view sketch and dimensions of the samples prepared via inkjet-printing of MWCNT ink on an ETFE substrate. The striped outer edges correspond to the areas glued onto the tensile machine holders.

**Figure 2:**
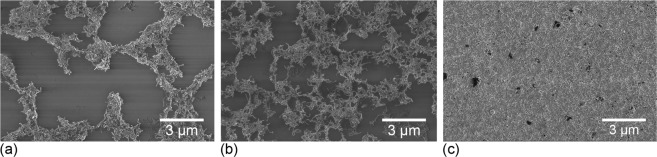
High-resolution SEM images of the (**a**) 2-layer, (**b**) 6-layer and (**c**) 20-layer samples.

An MWCNT ink is deposited via inkjet-printing as a 1.6 mm by 1.6 mm square pattern at the centre of a 125 µm-thick ETFE substrate measuring 18.7 mm by 6 mm (see Fig. 1 and Methods Section for more details). As described in detail in Michelis *et al*.^8^, MWCNT jetting from the printer head results in micrometric drops of entangled MWCNTs spread across the substrate according to a predefined pattern. To achieve connectivity between the drops and thus electrical percolation, several passes of the printer head over the same area are required. In the rest of this paper, a “layer” refers to the MWCNT deposition resulting from one pass of the printer head. Three samples bearing 2, 6 and 20 layers with thicknesses of 108 nm, 198 nm and 1,080 nm, respectively, (see Fig. 2) are prepared and each submitted to a tensile test within an SEM (see Methods Section for more details) at a displacement rate of 0.2 µm.s^−1^ in order to reach an average target strain of 0.5% at the substrate scale.

**Figure 3:**
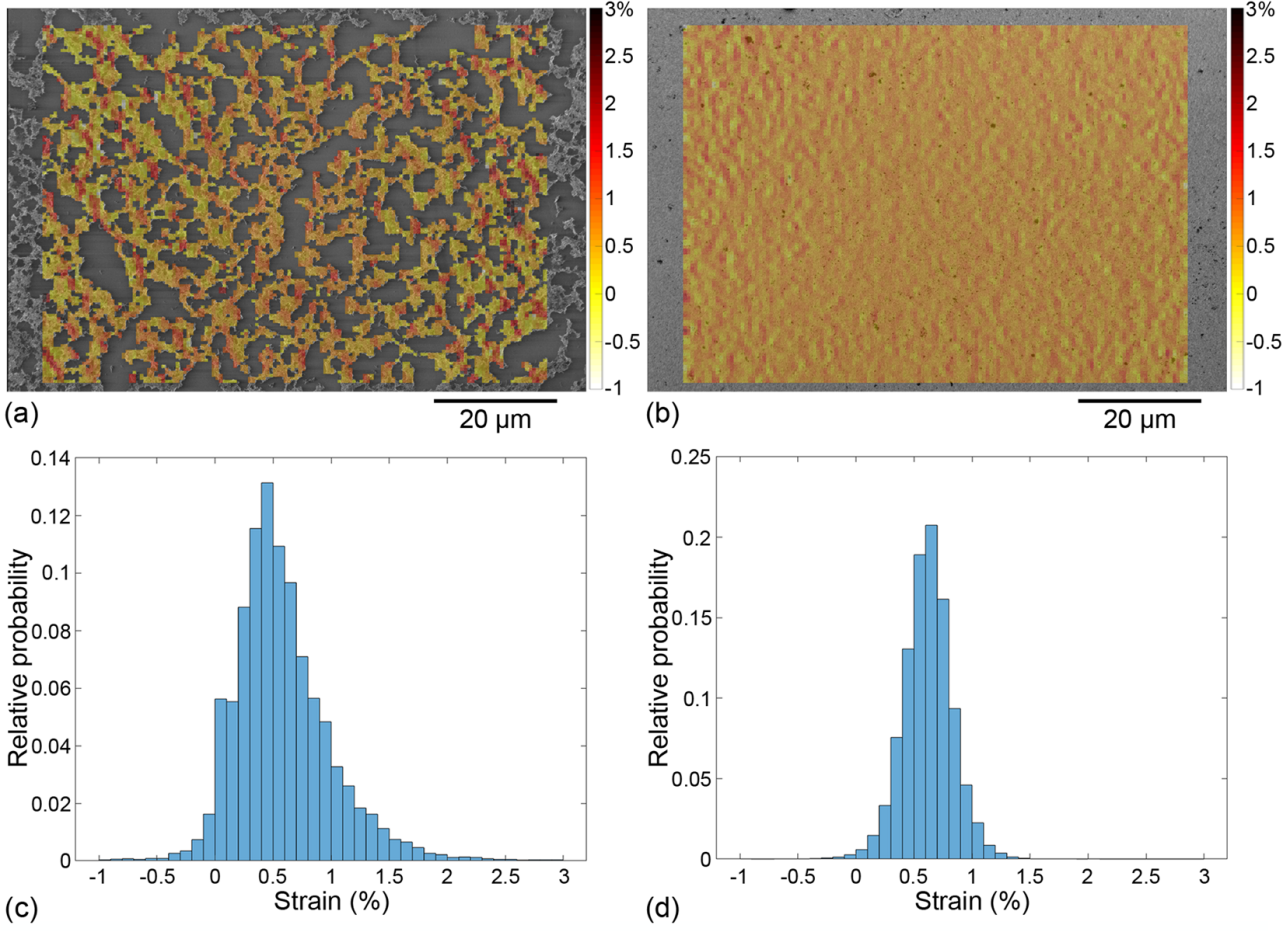
Longitudinal strain fields for low-resolution images corresponding to (**a**) 2 layers and (**b**) 20 layers. Histograms of strain field value distributions plotted for (**c**) 2 layers and (**d**) 20 layers.

**Figure 4:**
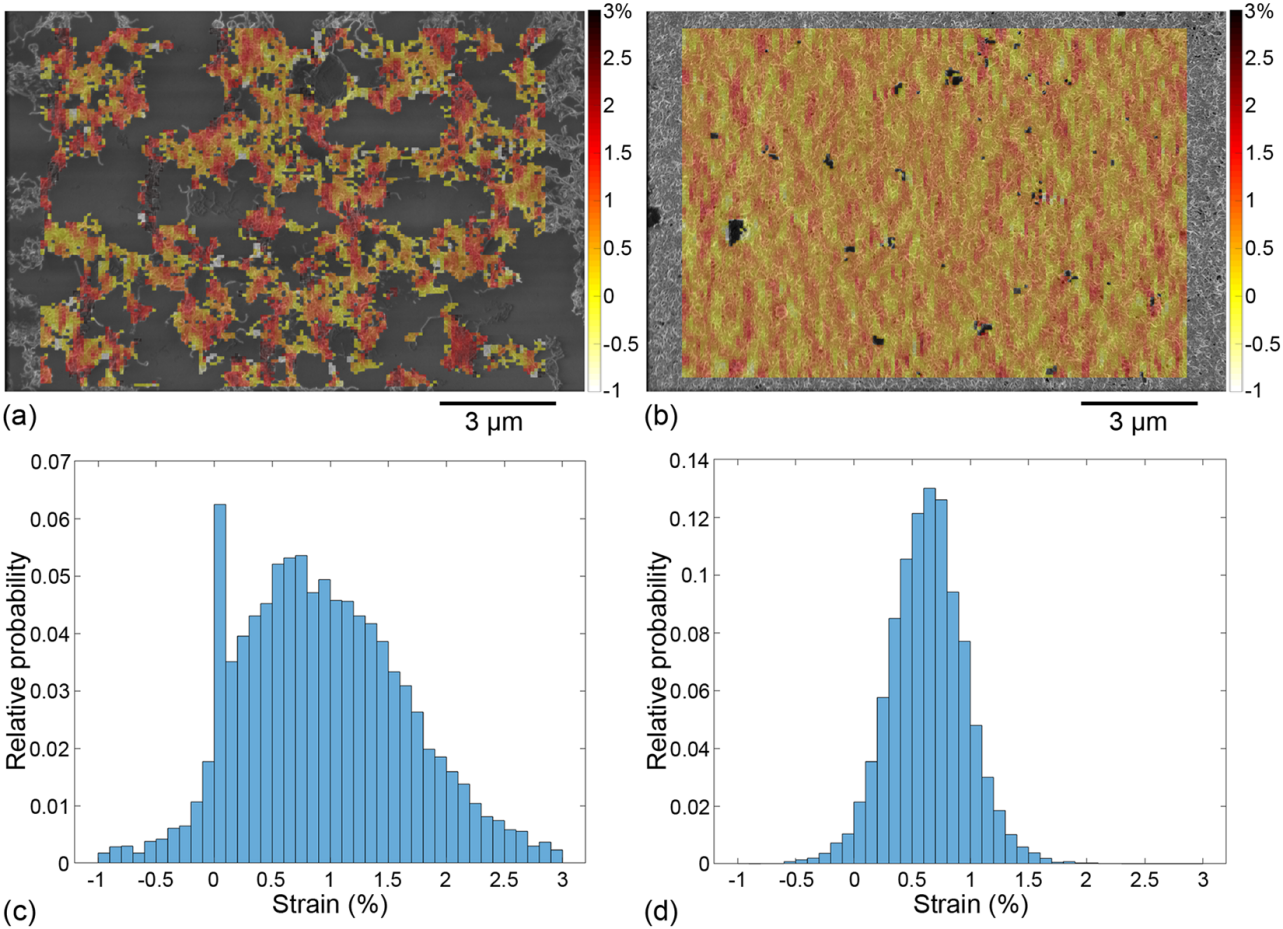
Longitudinal strain fields for high-resolution images corresponding to (**a**) 6 layers and (**b**) 20 layers. Histograms of strain field value distributions plotted for (**c**) 6 layers and (**d**) 20 layers.

**Figure 5:**
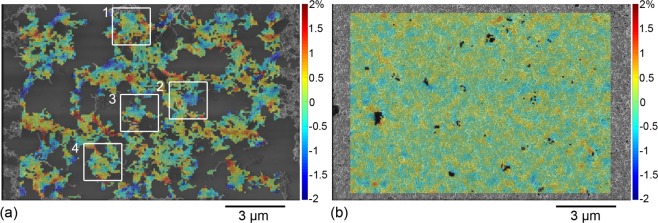
Shear strain fields for high-resolution images corresponding to (**a**) 6 layers and (**b**) 20 layers.

Images of the samples at rest and under the target strain are taken. For the 20-layer sample, one location is tracked at low magnification and another at high magnification. The 2- and 6-layer samples are each tracked in one location at low and high magnification, respectively. The images are subsequently processed by DIC. In this work, the grey-level contrasts of sub-micron features within the CNNs are exploited for the first time to serve as a speckle in order to access motions at the scale of CNTs. This processing yields the values of strain induced by the applied loading at the top of the MWCNT deposition. More details about the DIC processing used here as well as the method to estimate uncertainty on strain are provided in the Methods Section and in the Supplementary Information, respectively. For display and interpretation, the values of strain are projected in the reference frame and overlaid onto the SEM image of the CNN at rest (see Figs 3a,b, 4a,b and 5). In all cases, the tensile direction corresponds to the horizontal axis (**e**_1_).

### Strains in the substrate

The ETFE substrate is expected to behave as a linear isotropic elastic thin film: under tensile loading, its longitudinal strains (Green-Lagrange strain component E_11_) should be homogeneous and its shear strains (Green-Lagrange strain component E_12_) should be zero across the probed area (details about Green-Lagrange strain components can be found in the Supplementary Information). Note that independent tensile tests were conducted on the substrate alone to confirm that its overall transformation could actually be considered homogeneous (see Supplementary Information).

When the substrate can be seen in the SEM images, such as in the case of the 2- and 6-layer depositions (Fig. 2a,b, respectively), the overall longitudinal strain of the substrate (referred to as “substrate mean strain”) can be computed by following the motion of at least 3 dust specks attached to the substrate and assuming the overall transformation is homogeneous. This yields a strain of 0.51% in the case of the 2-layer image (low resolution) and of 0.56% in the case of the 6-layer image (high resolution). These are expected based on the calibration of the tensile machine (see details on tensile machine calibration in the Methods Section). Note that in the case of 20-layer deposition, the substrate is fully covered by CNTs (Fig. 2c) and hence the substrate mean strain cannot be accessed in the same manner.

### Strains in the MWCNT networks

Longitudinal strain fields and the corresponding strain histograms are plotted for all low-resolution images in Fig. 3 and for all high-resolution images in Fig. 4. Table 1 provides the statistics of longitudinal strain values (in the various configurations) in addition to the substrate mean strain—when it can be computed—and to the uncertainty on DIC strain data.

**Table 1:** Substrate mean strain, uncertainty on DIC strain and values of mean and standard deviation for the histograms presented in Figs 3 and 4.

*Number of layers*	Low-resolution images		High-resolution images	
	2	20	6	20
Substrate mean strain [%]	0.51	N/A	0.56	N/A
Uncertainty on DIC strain [%]	0.22	0.18	0.46	0.59
Histogram mean strain [%]	0.58	0.62	0.95	0.64
Histogram absolute standard deviation on strain [%]	0.44	0.21	1.0	0.33

Longitudinal strains maps are much more heterogeneous in thinner (and sparser) CNN depositions (Figs 3a and 4a) than in thicker (and denser) CNN depositions (Figs 3b and 4b). Histograms for the 2- and 6-layer samples (Figs 3c and 4c, respectively) indeed show that the spread of the strain values between the minimum and the maximum is several times larger than the uncertainty on DIC strain, contrary to the 20-layer sample at both resolutions (Figs 3d and 4d). Hence the 2- and 6-layer depositions exhibit local strains that clearly deviate from the substrate mean strain. This is further confirmed by the computation of the absolute standard deviation of the strain distributions (see Table 1). For comparable measurement uncertainty between the 2-layer and 20-layer samples at low resolution, the absolute standard deviation on the longitudinal strain is twice larger than the uncertainty in the case of the 2-layer sample (indicating a heterogeneous strain field) and it is of the same order of the uncertainty in the case of the 20-layer sample (indicating a nearly homogeneous strain field). The observation is identical for high-resolution data since the absolute standard deviation is twice larger than the uncertainty in the case of the 6-layer sample and even lower than the uncertainty in the case of the 20-layer sample.

To get further insight into the behaviour of the CNNs at the CNT scale, the shear strain maps for high-resolution images are now plotted in Fig. 5. Comparable to what was observed for the longitudinal strains, heterogeneities arise in the shear strains beyond the uncertainty on DIC strain in the 6-layer sample (Fig. 5a) with a standard deviation of 0.55%, whereas for the 20-layer case (Fig. 5b), the values are more homogeneous around zero with a standard deviation of 0.24% below uncertainty on DIC strain.

Overall, the analysis of longitudinal and shear strains shows that the motion of the MWCNTs at the top of the 2- and 6-layer samples (thinner and sparser CNN depositions) is heterogeneous and therefore significantly different from the substrate homogeneous motion. This suggests complex CNT motions in the thin and sparse depositions as well as imperfect and non-uniform substrate adhesion. On the contrary, the heterogeneities in the 20-layer sample remain comparable or smaller than the strain measurement uncertainties, indicating a more homogeneous motion of the MWCNTs at the top of this thicker and denser CNN deposition. This further implies that when CNTs are densely packed and form a thick layer, their overall motion resembles more closely the behaviour of the substrate.

### Deciphering local motions in thin and sparse CNN depositions

**Figure 6:**
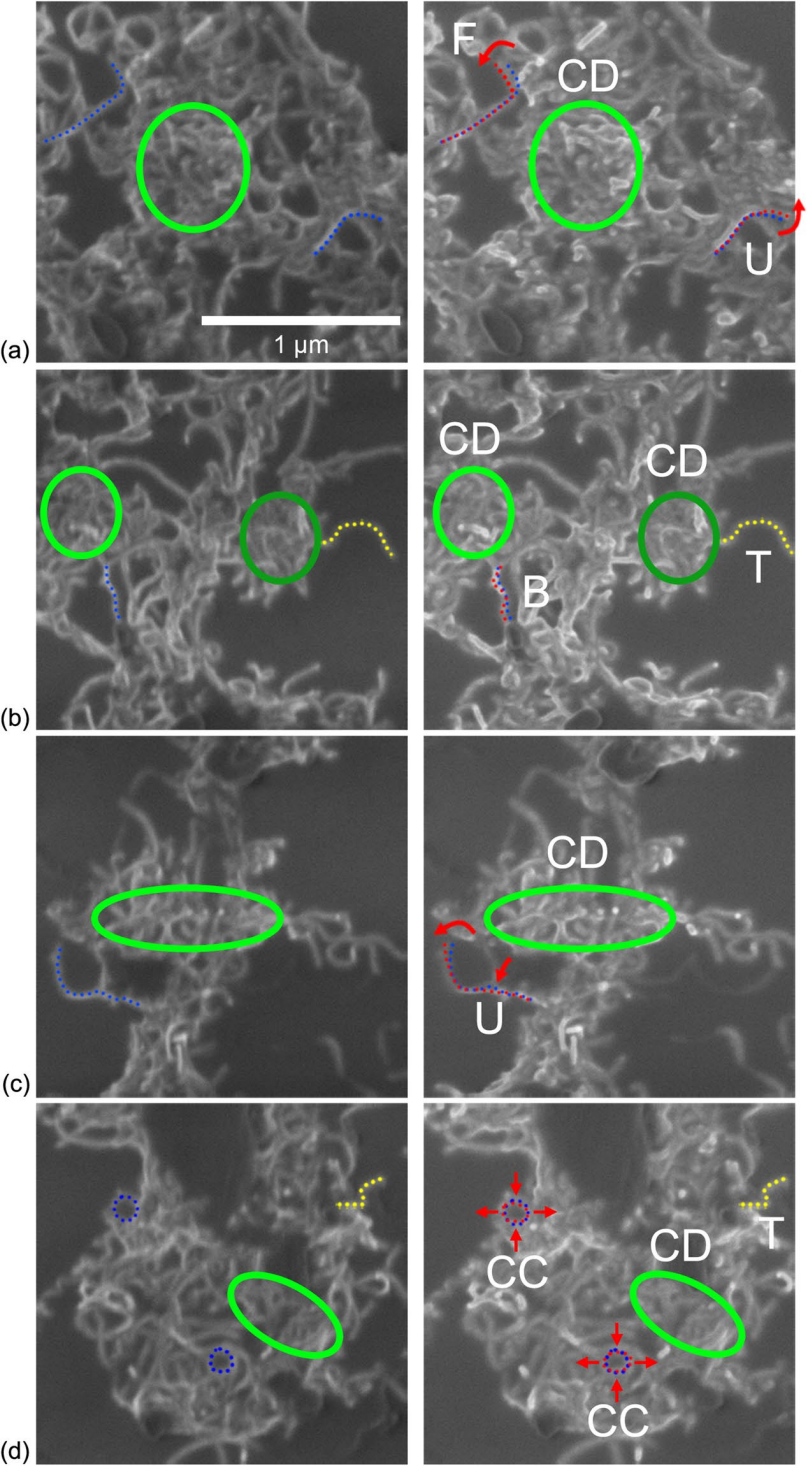
Initial (left) and deformed (right) configuration images in the specific areas highlighted in Fig. 5a: (**a**) area #1, (**b**) area #2, (**c**) area #3 and (**d**) area #4.

Non-zero shear strains are the sign of local gliding movements within the CNN, and strongly varying shear strains (proximity of high and low values of shear, i.e. red and blue values in the colour scale, respectively—see examples in Fig. 5a) indicate areas that are subjected to high relative gliding movements. To better understand the mechanisms of CNT motions at the CNT scale in the 6-layer deposition, focus is specifically set on four representative areas (highlighted in Fig. 5a) containing both strongly varying shear strains and homogeneous strains. For these selected areas, the corresponding images of the initial and deformed configurations are plotted in Fig. 6 (left and right, respectively) and the shear and longitudinal strains are plotted in Supplementary Information Fig. S4. A closer look at these areas uncovers, in what follows, key mechanisms arising in the corresponding CNN.

In all the selected areas, one can notice tight aggregates of CNTs: they are circled in green in Fig. 6. These aggregates all exhibit rather low homogeneous shear strains and homogeneous longitudinal strains. Hence there is little to no relative gliding motion between the CNTs constituting these tight aggregates; they remain tightly packed during deformation and exhibit a collective deformation, denoted CD in Fig. 6a–d. One notes, however, that the longitudinal strain in these aggregates is higher than the substrate mean strain and it remains unclear thus far as to what parameters control the deformation intensity within these aggregates.

One also observes looser aggregates in which individual CNTs form cell-like structures. In loosely packed aggregates, the cells generally tend to expand along the tensile direction and to contract along the transverse direction. This effect is highlighted here on two small and initially round cells marked CC in Fig. 6d: at the top left and bottom, circular cells become ellipses by elongating in the tensile (horizontal) direction and contracting in the transverse (vertical) direction, as marked by the red arrows. Note that this cell closing process does not lead to strong variations in the shear strains map nor in the longitudinal strains map.

Beside aggregates, one can identify individual CNTs attached to them. The strongly varying shear strains actually appear to concentrate around such CNTs. When comparing the high-resolution SEM images before and after loading, one observes that the individual CNTs undergo complex deformation mechanisms. The CNT marked F in Fig. 6a initially features two branches making a 45° angle pointing in the tensile direction (top left in Fig. 6a). Each branch appears to connect two CNT aggregates. After deformation, the CNT has folded further, as can be seen by comparing its initial position (blue dots) with its new position (red dots) with the folding direction highlighted by the red arrow. On the contrary, the CNTs marked U, located at the bottom right in Fig. 6a and at the bottom left in Fig. 6c, appear to unwind. Again, the initial configurations of the CNTs are marked with blue dots and their new configurations with red dots, while the red arrows highlight the unwinding direction. The vertical CNT marked B that appears to connect two aggregates (middle in Fig. 6b) buckles as the aggregates get closer to each other along the transverse (vertical) direction. The new shape, highlighted by the red dots, is significantly wavier than the initial shape marked by the blue dots. For all the above-mentioned locations, strongly varying shears (presence of very different colours) seem to arise in their vicinity but do not necessarily lead to heterogeneities in the longitudinal strain maps.

Hence individual CNTs that are linked to different aggregates deform so as to accommodate the general motion of these surrounding aggregates, i.e., extension in the tensile (horizontal) direction and compression in the transverse (vertical) direction due to the Poisson effect arising in the supporting substrate. This leads to different responses according to their initial morphology and orientation. In contrast to the CNTs that exhibit these folding, unwinding or buckling motions, some CNTs feature only a translation movement independently of their initial orientation; they are marked as T and highlighted with yellow dots (right in Fig. 6b,d). These CNTs appear to be connected only very loosely to aggregates (usually on one side only in a manner that seems to emerge from the aggregates).

**Figure 7:**
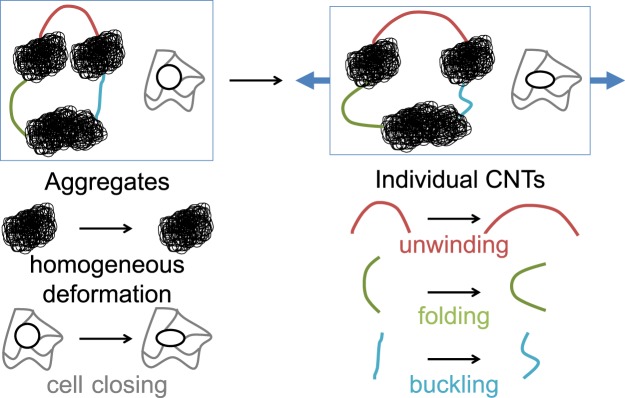
Sketch of the deformation mechanisms observed in thin and sparse CNNs.

To summarize these observations regarding CNT motions in thin and sparse CNNs, CNT aggregates appear to feature little strain heterogeneity as they exhibit either a collective deformation for the tighter ones or a cell-closing process for the looser ones. Individual CNTs loosely linked to aggregates (isolated or connected only on one side) tend to only feature translational motions. Individual CNTs connecting aggregates tend to unwind when mainly oriented in the tensile (horizontal) direction and to fold or buckle (depending on their initial morphology) when mainly oriented in the transverse (vertical) direction. Such motions of individual CNTs lead to strong variations in the shear strain field. These uncovered mechanisms are also summarized as a sketch in Fig. 7.

The different behaviour within the two types of aggregates may be explained by differences in the strength of the CNT-to-CNT interactions. These interactions are of Van der Walls types and so are strongly dependent on the CNT-to-CNT distance and on the surface area of interaction. In tight aggregates, CNT-to-CNT distance is expected to be reduced, and CNTs are in contact with other CNTs over their whole outer surface. As such, they are tightly bound together by Van der Walls forces. One expects this to prevent internal reorganizations of the aggregate which then roughly behaves as a rigid body. By contrast, in loose aggregates, the Van der Walls interactions are expected to be less intense due to the increased CNT-to-CNT distance and to the reduced surface area of contact. This would explain the internal reorganization observed within such aggregates.

### Mechanisms of motion and modelling insight for CNN depositions

The following mechanisms of motion are thus proposed for thin and sparse CNN depositions: both the tight and loose aggregates deform homogeneously whereas individual CNTs that are strongly attached to these aggregates deform to accommodate their motion (hence causing the higher variability in the strain fields). On the other hand, thick and dense CNNs exhibit relatively homogeneous strains: within the 20-layer CNN, no individual CNT can be singled out and it appears exclusively to be made of tight and loose aggregates (Fig. 2c).

In order to correlate these local behaviours to macroscopic features of the film such as piezoresistivity and Young’s modulus, the use of numerical multiscale models of CNN^21^ would be needed. This is beyond the scope of the present study. Nevertheless, for accurate modelling, the reported observations suggest that models of sparse CNNs—notably those featuring aggregates—should at least account for heterogeneous strain fields on the top layer of the deposition. Furthermore, those sparse CNNs could be interestingly modelled, for example, as multiphase media^27^ with at least one—possibly two—aggregate phases (tight and loose aggregates) and one—possibly two—phases with individual CNTs (loosely and tightly connected CNTs). It would drastically reduce computation times compared to methods treating all CNTs as individual^39^.

Finally, since strain heterogeneities are observed on the top layer of thin and sparse CNN depositions while the substrate undergoes homogeneous strains, the adhesion between the substrate and the CNN layer is expected to be non-uniform. Some parts of the CNN may even detach from the substrate. Such imperfect substrate adhesion is already accounted for in models of strain release in CNNs, where it explains buckling effects^29^. The present results suggest it should also be integrated in models describing tensile loadings.

## Conclusion

This study constitutes the first *in-situ* observation of CNNs of various thicknesses under tensile strain at a scale where CNT aggregates as well as individual CNTs can be identified. This is also the first time that strain data is computed in inkjet-deposited MWCNT networks at such resolution and helps to understand the CNT motion mechanisms. Indeed, the obtained DIC data shows that the strains are heterogeneous at the top of thin and sparse depositions, while strains get more homogeneous at the top of thick and dense depositions. Heterogeneities in the shear strain fields at the top of the former depositions can be linked to individual CNTs that fold, unwind, buckle or simply translate according to their orientation versus tension as well as their connectivity to the surrounding aggregates. Aggregates, on the other hand, show more homogeneous strains even though the mechanisms at hand are different: tightly packed aggregates exhibit a collective deformation whereas loosely packed ones behave like a foam through the closing of their cell-like features. Overall, the results suggest that the behaviour of thin and sparse CNN depositions is controlled by aggregates while individual CNTs accommodate their motion. There is also a strong possibility of imperfect and non-uniform substrate adhesion. The uncovered features confirm that consideration of multiphasic models, as hypothesized in Yin *et al*.^26^, would constitute valuable additions to achieve realistic mechanical models of substrate-supported CNNs. Finally, since there are now means to control both the size and density of aggregates in CNN composites^40^, these findings open the path towards an aggregate-mediated fine-tuning of CNN motion.

## Methods

### Fabrication of samples

The fabrication process for the CNN thin films is described in detail in Michelis *et al*.^8^. The CNTs used in this study are Graphistrength C100 MWCNTs from Arkema. These nanotubes are typically of dimensions 10–15 nm in diameter and 1–10 µm in length. MWCNTs are first dispersed in 1,2-Dichlorobenzene (DCBZ) at 0.02 wt.% using an ultrasonic probe (Bioblock Scientific VibraCell 75043) operated at 150 W for 20 min. After centrifugation (Heraeus Megafuge 8 R) at 8 kG for 10 min, the homogeneous phase of the liquid is extracted. After a second centrifugation at 11 kG for 4 h, the homogeneous phase of the liquid is again extracted. Sodium dodecyl benzene sulphonate (SDBS) at 0.3 wt% is added and the mixture is further dispersed using an ultrasonic probe operated at 150 W for 20 min in order to increase ink wettability and deposition homogeneity. The obtained ink is deposited as a 1.6 mm by 1.6 mm square pattern at the centre of a 125 µm-thick ETFE substrate 18.7 mm by 6 mm in size and purchased from Goodfellow (see Fig. 1). The deposition is made layer-by-layer via inkjet-printing with a 2800 Dimatix Material Inkjet Printer equipped with a 1 pL cartridge. After each layer, the substrate is dried at 55 °C for 10 min. A rinsing step is carried out every two layers by immersion and slight agitation in methanol and acetone for 8 s, each followed by drying under air flow. This rinsing step washes away residual DCBZ and SDBS and thus increases the conductivity of the printed layers. Three samples are thereby prepared, each respectively bearing 2, 6 and 20 layers (see Fig. 2). Due to the low density of CNTs on the surface, the 2- and 6-layer samples are well below the electrical percolation threshold (resistance larger than 50 MOhms) whereas the 20-layer sample is past the percolation threshold (resistance in the 150 kOhms range). Note that resistance measurements are made in a 4-probe configuration on separate samples so as not to damage the substrate with the probes prior to tensile testing. Details on the setup are reported in Michelis *et al*.^8^ along with a demonstration of the high reproducibility of the fabrication process and an estimation of the CNN thicknesses (108 nm, 198 nm and 1,080 nm for the 2-, 6- and 20-layers depositions, respectively).

### *In-situ* tensile SEM

**Figure 8:**
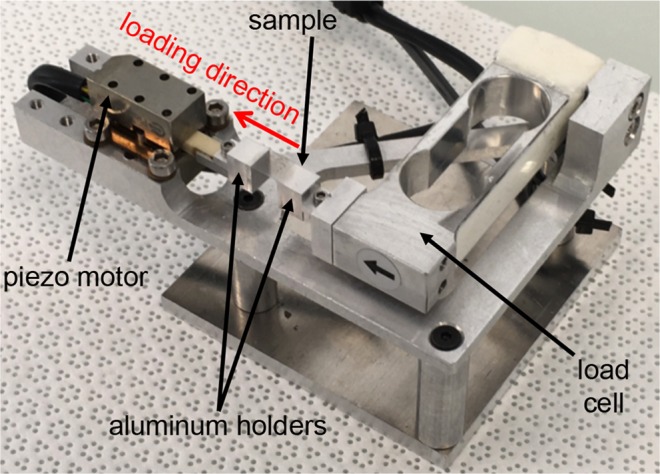
Custom-made tensile machine for *in-situ* tensile testing within an SEM.

*In-situ* tensile testing within an SEM chamber relies on a custom-made miniaturised tensile machine. It consists of an aluminium frame bearing a linear motor (LT2010D Piezo LEGS® Linear Twin 20 N non-magnetic vacuum version from PiezoMotor) and a 20 N load cell (LCAE from Omega), each facing each other. Both the motor and the load cell are equipped with aluminium holders having a top surface area of 6.1 mm by 6 mm (Fig. 8). Both ends of the sample are glued on top of these holders. The displacement of the motor yields a uniform tensile strain state where the CNT deposition lies. The tensile machine is mounted within a FEI Helios NanoLab 660 SEM. In order to reduce charging and drift effects, the SEM is operated with low accelerating voltage (500 V), low beam current (between 50 and 100 pA) and low working distance (4 mm). Images are acquired using the Through Lens Detector in polarization mode (with a stage bias in the range 50–100 V) and via integration of 32 scans taken with a dwell time of 500 ns. Additionally, the cold trap fed with liquid nitrogen is activated in order to attract impurities that may be floating in the chamber and thus ultimately to decrease the level of contamination during the scanning of the sample.

Before performing tests within the SEM, the tensile machine needs to be calibrated specifically for the studied sample size. Indeed, the Piezo LEGS^®^ motor used in this study relies on a stick-slip technology where electrical impulsions cause piezoelectric legs to impart, as they bend back and forth, a displacement by friction on the yellow ceramic driving rod (see Fig. 8). This displacement depends on the pullback force applied to the rod while friction operates, which in turn depends on the overall stiffness of the sample that is mounted on the tensile system. A calibration of the system is thus conducted on 8 different and bare ETFE samples of dimensions reported in Fig. 1. The motor is set to perform a displacement from 0 to 500 µm at 0.2 µm.s^−1^ while the local strain in the freestanding part of the ETFE substrate is measured by optical extensometry (see Bodelot *et al*.^41^ for details). This allows one to obtain the displacement needed for the motor to attain a given target strain in the substrate. It has been found that imparting a displacement of 254 µm leads to an average tensile strain of 0.5% in the ETFE substrate (with a minimum of 0.40% and a maximum of 0.68% over the 8 conducted tests). Within the SEM, each sample is then submitted to a tensile test with the motor operated at 0.2 µm.s^−1^ so as to reach the average target strain of 0.5% within the substrate. Note here that the CNT sensors under study have been shown to exhibit a reversible, linear, hysteresis-free behaviour up to a strain threshold of 0.07%^8^. However, since our goal is to investigate the deformation mechanisms within the CNT layers, the strain applied to the sample is purposely taken higher (being at most 10-fold of this threshold) so as to enhance the features of CNT motion while safely remaining within the reported linear elastic isotropic range of ETFE^42^. Images of the samples at rest and under the target strain are taken at either low (image width 95 µm and pixel size close to 31 nm) or high magnification (image width 15 µm and pixel size close to 5 nm). For the 20 layers, two locations are tracked under target strain: one at low magnification and the other at high magnification. Note that for the 2- and 6-layer samples, since the CNT films are below the electrical percolation threshold and ETFE is a dielectric, some charging and drift effects still occur despite the above-mentioned precautions—in particular for the 2-layer sample at high magnification. Hence, the 2- and 6-layer samples are tracked at low and high magnification, respectively.

### Digital image correlation

DIC is an image processing technique that can be applied to two-dimensional images exhibiting a wide range of randomly distributed grey levels^43^. The reference image, corresponding here to the sample at rest, is first divided into square regions called subsets. As each subset has a particular distribution of grey levels, it (and thus its position) can be tracked in subsequent images of the deformed sample. This yields the in-plane displacement fields within the analysed area, from which the strain fields can be derived. Comprehensive details about the DIC technique can be found in Sutton *et al*.^44^. The software used in this study is CorrelManuV^45,46^. Even though DIC is regularly used to analyse strain fields at the macroscopic scale in bulk materials (polymers, cement) enhanced with carbon-nanotubes^47–50^, only Maschmann *et al*.^37^ so far have tackled the application of DIC to a CNN (namely, to vertically aligned 30 μm-wide and 75 μm-tall pillars made of CNTs). However, the resolution of the method does not reach the CNTs themselves. In the images obtained herein, whatever the magnification, the CNT depositions show contrasted grey levels, whereas the substrate—when visible—corresponds to a rather uniform shade of grey. The present paper thus proposes the application of DIC to sub-micron features exhibiting contrasted grey levels within the CNN in order to access motions at the scale of CNTs. Hence, once the initial grid of subsets is generated for the whole image, subsets falling onto the substrate are identified and excluded from the correlation process. This concerns all high-resolution images as well as the 2-layer sample imaged at lower resolution and it explains why some strain fields presented in Section 2 appear sparse. The subset size is taken as 30 × 30 pixels on low-resolution images and 80 × 80 pixels on high-resolution images, thus yielding the respective resolutions of 928 nm and 391 nm for a subset. Note that the choice of subset size derives from a compromise between precision and resolution of the strain fields (a larger subset leads to better precision but to lower resolution). Additional information regarding DIC uncertainty estimates can be found in the Supplementary Information; the uncertainty on strain is reported in Table 1.

## Supplementary information

Supplementary Information

## Data Availability

The datasets generated and analysed during the current study are available from the corresponding author on reasonable request.
