# Supplementary material for: Aggregate-driven reconfigurations of carbon nanotubes in thin networks under strain: in-situ characterization
Source: Sci Rep. 2019 Apr 2;9:5513. doi: 10.1038/s41598-019-41989-2 (PMC6445119; doi:10.1038/s41598-019-41989-2)
Supplement: Supplementary file 1 — Supplementary Information [file 41598_2019_41989_MOESM1_ESM.pdf]

Supplementary Information

Aggregate-driven reconfigurations of carbon nanotubes in thin networks under strain: in-situ characterization

Laurence Bodelot[1,*], Luka Pavić[1,^], Simon Hallais[1], Jérôme Charliac[2], Bérengère Lebental[2,3]

1. Ecole Polytechnique, Laboratoire de Mécanique des Solides (LMS), 91128 Palaiseau, France

2. Ecole Polytechnique, Laboratoire de Physique des Interfaces et Couches Minces (LPICM), 91128 Palaiseau, France

3. Université Paris-Est, IFSTTAR, COSYS, Marne-La-Vallée 77447, France

^Current affiliation: Division of Materials Chemistry, Ruđer Bošković Institute, Bijenička cesta 54, 10000 Zagreb, Croatia

*Corresponding author: laurence.bodelot@polytechnique.edu, +33 1 69 33 57 46

DIC uncertainty estimates

Two types of errors usually arise when performing DIC: the systematic error and the random error. To summarize, the systematic error is inherent to the interpolation embedded in the DIC sub-pixel correlation algorithm[1,2] while the random error is linked to contrast variations due to the imaging process[3,4]. The impact of the systematic error is evaluated by performing correlation (with different subset sizes) on a sequence of images that have been numerically shifted from 0 to 1 pixel by 0.1 increments. Firstly, the standard deviation in the displacement field data is computed as a function of the shift (the average of the field data corresponds to the imparted shift) for the considered subset sizes. Then, the displacement uncertainty is defined as the average of the above-mentioned standard deviations and plotted as a function of the subset size. The impact of random error is obtained by performing correlation (with different subset sizes) on two images of the same scene. The standard deviation in the displacement field data is retrieved (the average should be 0 since there is no displacement between the two images) and yields the displacement uncertainty as a function of the subset size. This helps in considering the compromise that is made between uncertainty and the resolution that depends on the subset size. As an illustrative example, results obtained on the 20 layers low- and high-resolution images regarding the systematic and random error are given in Fig. S1 and S2, respectively. As mentioned in the main text, a larger subset lowers the uncertainty to the detriment of spatial resolution.

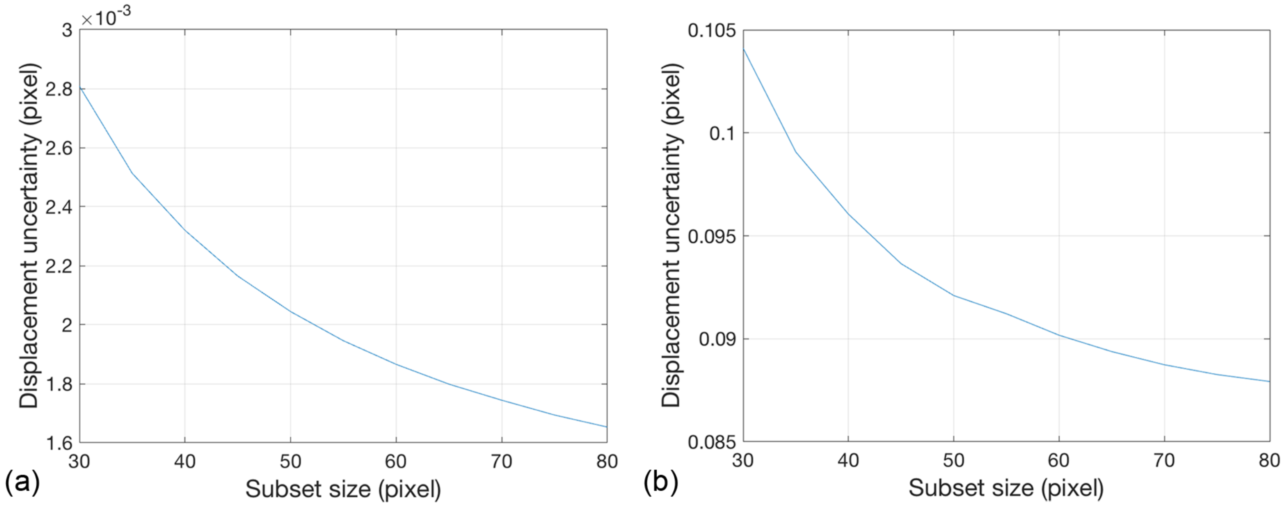

Figure S1. Case of the 20-layer low-resolution image: displacement uncertainty as a function of the subset size induced by **(a)** the systematic error and **(b)** the random error.

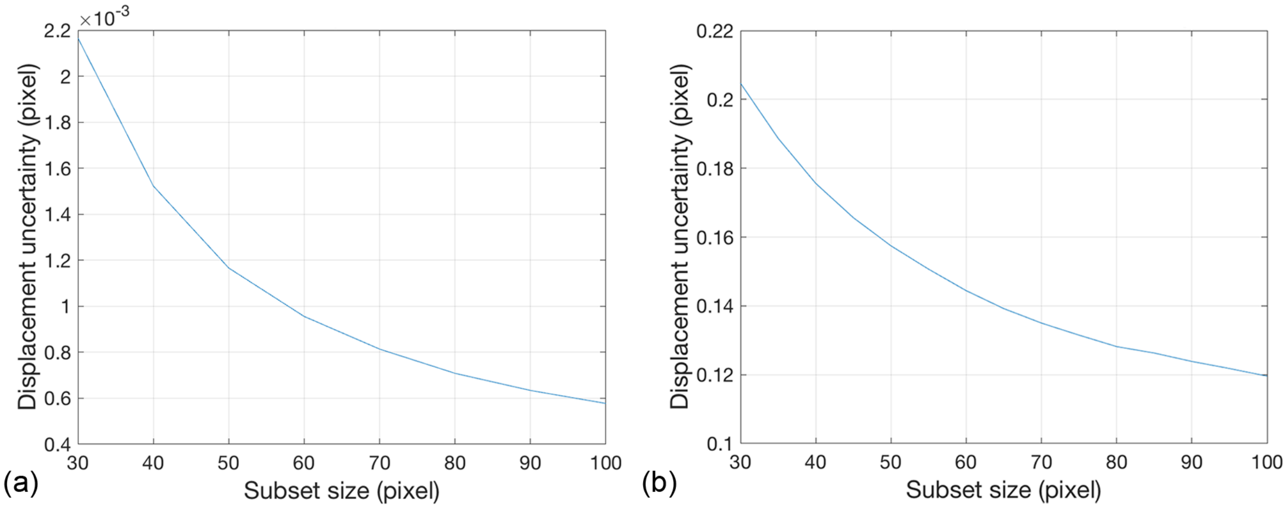

Figure S2. Case of the 20-layer high-resolution image: displacement uncertainty as a function of the subset size induced by **(a)** the systematic error and **(b)** the random error.

For the low-resolution images, where the subset size is taken as 30 pixels, the systematic error and random error yield a displacement uncertainty of 2.8×10^{-3} and 1.0×10^{-1} pixel, respectively. For the high-resolution images, where the subset size is taken as 80 pixels, the systematic error and random error yield an uncertainty on displacement of 7.1×10^{-4} and 1.3×10^{-1} pixel, respectively. Hence, in both cases, the uncertainty on displacement arising from the systematic error is much smaller than the uncertainty arising from the random error. This indicates that, with the images we are working on, the predominant error is linked to image quality.

The procedure described for the displacement field is repeated for the strain field (whose average should be zero since one considers here a rigid body motion) and yields the strain uncertainty as a function of the subset size. Strain uncertainty linked to the random error is thereby computed and reported in Table S1 for all sets of images considered in this study.

	Low-resolution images		High-resolution images	
Number of layers	2	20	6	20
Strain uncertainty [%]	0.22	0.18	0.46	0.59

Table S1. Error estimates for the images analyzed in this study.

Strain components

In an orthonormal coordinate system of axes ($\mathbf{e}_1$, $\mathbf{e}_2$, $\mathbf{e}_3$), let us denote **X** the position (X_1, X_2, X_3) of a material point in the reference configuration of a continuous medium and **x** its position (x_1, x_2, x_3) in the deformed configuration. In mechanics, a homogeneous transformation between the reference and the deformed configuration is described by the gradient of the transformation $\mathbf{F}=\partial\mathbf{x}/\partial\mathbf{X}$, where **F** is a second-order tensor. One also usually computes the right Cauchy-Green tensor $\mathbf{C}=\mathbf{F}^T.\mathbf{F}$, where subscript T indicates the transposition. Diagonal components of **C** correspond to the square of the dilation of vectors that were initially along the basis vectors (e.g. vectors $\mathbf{V}_1$ and $\mathbf{V}_2$ in Fig. S3), while off-diagonal components of **C** are related to angle variations (due to shear) between the basis vectors. For example, $C_{12}/(C_{11}C_{22})^{1/2}$ is the cosine of the angle θ (in the deformed configuration) between vectors (e.g. $\mathbf{v}_1$ and $\mathbf{v}_2$) that were initially along $\mathbf{e}_1$ and $\mathbf{e}_2$. Information on the variations of scalar products due to the transformation are finally obtained by computing the Green-Lagrange deformation tensor **E** as 1/2.(**C**-**I**), where **I** is the unit tensor. Its diagonal components are a measure of the strain underwent by vectors that were initially along the basis vectors and its off-diagonal components are a measure of the shear between these vectors.

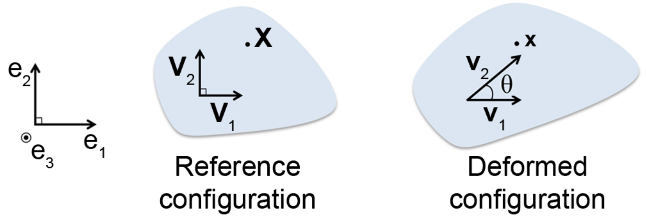

Figure S3. Sketch depicting the transformation of elementary vectors between the reference and deformed configurations.

Substrate deformation

A piece of substrate having the same dimensions than the samples (see Fig. 1 in the main manuscript) is submitted three times for 5 min to the plasma of the SEM used in this study so as to render it hydrophilic[5,6]. A water solution containing latex nanobeads 280 nm in size at 10 wt.% is drop-casted onto the sample and left to dry in air. A 15 nm layer of gold is then deposited by sputtering and the sample cleaned for 20 min in water in an ultrasonic bath. This process enables to create a gold speckle pattern when the nanobeads are washed away and gold remains at the locations that were not covered by the beads. The speckled sample is then submitted to an in-situ tensile test in the same conditions than described in the Experimental Section and images of the sample at rest and under strain are processed by DIC. Imaging conditions in the SEM are similar to what was

used for the CNN samples (i.e. low voltage, low working distance and low pressure), the magnification is so that the field of view is 95 µm wide (i.e. pixel size close to 31 nm) and the subset size in correlation is 30 x 30 pixels. Error estimates were computed for this subset size following the procedure described in Section S1. The uncertainty on displacement caused by the systematic and random errors are 4.1×10^{-3} and 4.8×10^{-1} pixel, respectively. Hence, the largest uncertainty on strains come from the random error and is 0.14%. The longitudinal strain field is then computed and yields a standard deviation of 0.13% for an average strain of 0.60%. The fact that the standard deviation remains below the uncertainty confirms that the ETFE substrate deforms homogeneously at the probed scale.

Shear and longitudinal strains in the four representative areas

In the main manuscript, we focused on the initial and deformed configurations of four representative areas (highlighted in Fig. 5a) containing both strongly varying shear strains and homogeneous strains. The shear and longitudinal strains of these areas are reported in Fig. S4.

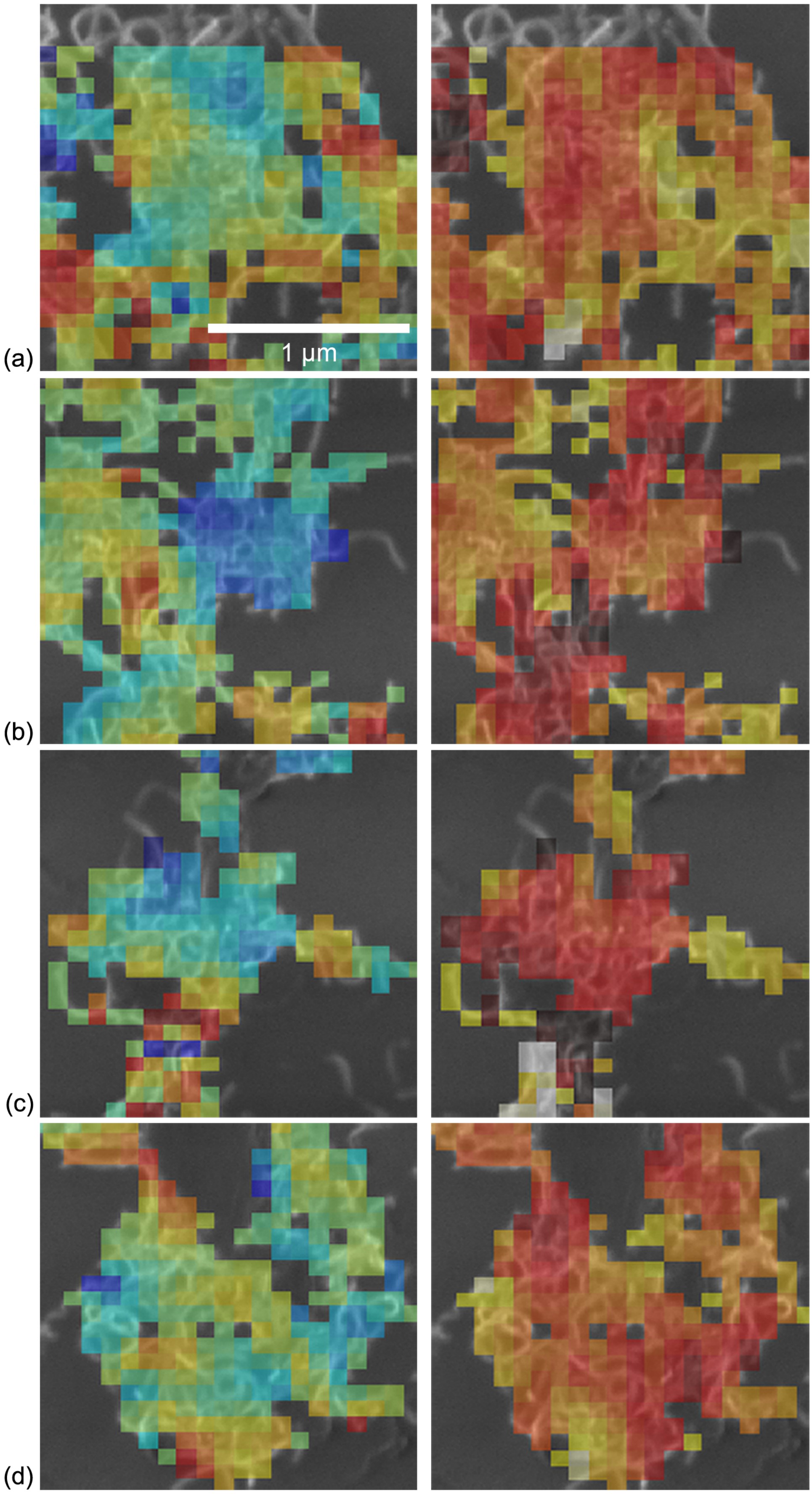

Figure S4. Left: shear strain maps, right: longitudinal strain maps, in the specific areas highlighted in Fig. 5a: **(a)** area #1, **(b)** area #2, **(c)** area #3 and **(d)** area #4.

References

1. Schreier, H. W., Braasch, J. R. & Sutton, M. A. Systematic errors in digital image correlation caused by intensity interpolation. *Opt. Eng.* **39,** 2915–2921 (2000).
2. Hild, F. & Roux, S. Digital Image Correlation: from Displacement Measurement to Identification of Elastic Properties - a Review. *Strain* **42,** 69–80 (2006).
3. Bornert, M. *et al.* Assessment of Digital Image Correlation Measurement Errors: Methodology and Results. *Exp. Mech.* **49,** 353–370 (2009).
4. Sutton, M. A., Orteu, J.-J. & Schreier, H. W. *Image Correlation for Shape, Motion and Deformation Measurements. Basic Concepts, Theory and Applications*. (Springer, 2009). doi:10.1007/978-0-387-78747-3
5. Bodas, D. & Khan-Malek, C. Formation of more stable hydrophilic surfaces of PDMS by plasma and chemical treatments. *Microelectron. Eng.* **83,** 1277–1279 (2006).
6. Lee, S., Park, J.-S. & Lee, T. R. The Wettability of Fluoropolymer Surfaces: Influence of Surface Dipoles. *Langmuir* **24,** 4817–4826 (2008).
